# Comparison of Indicators of Dependence for Vaping and Smoking: Trends Between 2017 and 2022 Among Youth in Canada, England, and the United States

**DOI:** 10.1093/ntr/ntae060

**Published:** 2024-03-26

**Authors:** Makenna N Gomes, Jessica L Reid, Vicki L Rynard, Katherine A East, Maciej L Goniewicz, Megan E Piper, David Hammond

**Affiliations:** School of Public Health Sciences, University of Waterloo, Waterloo, ON, Canada; School of Public Health Sciences, University of Waterloo, Waterloo, ON, Canada; School of Public Health Sciences, University of Waterloo, Waterloo, ON, Canada; Department of Addictions, Institute of Psychiatry, Psychology, and Neuroscience, King’s College London, London, UK; Department of Health Behavior, Roswell Park Comprehensive Cancer Center, Buffalo, NY, USA; Center for Tobacco Research and Intervention, School of Medicine and Public Health, University of Wisconsin Madison, Madison, WI, USA; School of Public Health Sciences, University of Waterloo, Waterloo, ON, Canada

## Abstract

**Introduction:**

The current study sought to examine trends in indicators of dependence for youth vaping and smoking during a period of rapid evolution in the e-cigarette market.

**Aims and Methods:**

Data are from repeat cross-sectional online surveys conducted between 2017 and 2022 among youth aged 16–19 in Canada, England, and the United States (US). Participants were 23 145 respondents who vaped and/or smoked in the past 30 days. Four dependence indicators were assessed for smoking and vaping (perceived addiction, frequent strong urges, time to first use after waking, days used in past month) and two for vaping only (use events per day, e-cigarette dependence scale). Regression models examined differences by survey wave and country, adjusting for sex, age, race, and exclusive/dual use.

**Results:**

All six indicators of dependence increased between 2017 and 2022 among youth who vaped in the past 30 days (*p* < .001 for all). For example, more youth reported strong urges to vape at least most days in 2022 than in 2017 (Canada: 26.5% to 53.4%; England: 25.5% to 45.4%; US: 31.6% to 50.3%). In 2017, indicators of vaping dependence were substantially lower than for smoking; however, by 2022, youth vaping was associated with a greater number of days used in the past month (Canada, US), shorter time to first use (all countries), and a higher likelihood of frequent strong urges (Canada, US) compared to youth smoking.

**Conclusions:**

From 2017 to 2022, indicators of vaping dependence increased substantially. By 2022, vaping dependence indices were comparable to those of smoking.

**Implications:**

Indicators of vaping dependence among youth have increased substantially since 2017 to levels that are comparable to cigarette dependence among youth who smoke. Future research should examine factors underlying the increase in dependence among youth who vape, including changes to the nicotine profile and design of e-cigarette products.

## Background

Nicotine is the primary constituent responsible for promoting and sustaining dependence on tobacco products.^1^ Like conventional combustible cigarettes, electronic cigarettes (e-cigarettes) can deliver nicotine in a manner that can promote nicotine dependence.^2,3^

As with combustible cigarettes, vaping products can deliver high concentrations of nicotine, although the rate of nicotine delivery varies by device design, e-liquid formulation, and patterns of product use.^3–6^ Pharmacokinetic studies conducted among some nicotine salt-based e-cigarettes, such as JUUL, demonstrate similar nicotine delivery for experienced vaping as for conventional cigarette smoking.^1,6^ Several studies suggest that JUUL and other salt-based products may be associated with greater levels of vaping dependence^7–9^; however, the extent to which the increasing popularity of salt-based products has affected population-level measures of dependence remains unclear.

People who regularly vape e-cigarettes also exhibit many of the core indicators of dependence as people who smoke tobacco: cravings, urges to use e-cigarettes, withdrawal symptoms, and frequent use throughout the day and soon after waking.^10,11^ To date, most studies have examined nicotine dependence among current or former adult smokers. These studies generally report lower levels of dependence on e-cigarettes compared to combustible cigarettes.^2,12–16^ Few studies have examined vaping dependence among youth, which is important for understanding whether vaping will evolve into a long-term, established behaviour as younger people who vape transition into adulthood.^17^ Studying young people who vape also provides an opportunity to examine vaping dependence among previously nicotine-“naïve” consumers.^18,19^

Several studies have examined indicators of vaping dependence among youth using large population-based surveys. A national survey conducted in Great Britain in 2022 found that 34% of youth aged 11–17 who used e-cigarettes reported urges to vape.^20^ In the United States (US), a national survey conducted in 2020 among 13- to 24-year-olds found that 70% of participants who exclusively used e-cigarettes experienced at least one symptom of nicotine dependence.^21^ Several studies have also analyzed data from the US National Youth Tobacco Survey (NYTS). These studies indicate an increase in cravings to vape and other indicators of dependence since 2017 among US high school students—the period in which vaping prevalence increased along with the popularity of JUUL.^22–24^ It should be noted, however, that NYTS may be underestimating vaping dependence due to the question wording, which refers to “tobacco products” rather than “e-cigarettes” or “vaping.” This is a notable limitation given that youth view e-cigarettes as distinct from combustible cigarettes and other tobacco products.^25^

Comparing levels of dependence between vaping e-cigarettes and smoking tobacco products provides an informative point of comparison, given the well-established evidence base on dependence and abuse liability of tobacco products.^26^ To date, two studies have directly compared indicators of dependence for vaping versus smoking among youth. A cross-sectional analysis of Population Assessment of Tobacco and Health (PATH) data collected in 2019 found similar levels of dependence among youth who vaped daily and youth who smoked daily based on a 7-item dependence scale.^27^ A study by Hammond et al. (2021) compared dependence indicators among youth between 2017 and 2019 in Canada, England, and the US. The findings indicated increases in urges to vape, perceived addiction, and frequency of use (times per day and days used in the past month) in all three countries, as well as a narrowing gap between vaping and smoking dependence, although to a greater extent in Canada and the US compared with England.^28^ Overall, there are indications that vaping dependence may be increasing among youth, particularly in North America; however, there is a lack of data to address trends over time, as well as comparisons with dependence on smoking tobacco.

The current study extends the earlier findings of Hammond et al. (2021) to include additional survey waves and dependence indicators among youth in Canada, England, and the US. The current analysis examines trends between 2017 and 2022, which is a period of rapid evolution in the e-cigarette market, characterized by a shift toward products with salt-based nicotine e-liquids and higher nicotine concentrations. As noted above, this transition was initiated by JUUL and then widely adopted by other brands over this period.^9,28–31^ The primary objective of the current study was to examine trends in six separate indicators of e-cigarette dependence among youth who vape over this period: perceived addiction, frequent strong urges, time to first use after waking, days used in past month, use events per day, and scores on the e-cigarette dependence scale (EDS). The study also compared the first four of these indicators among youth who exclusively vaped e-cigarettes, exclusively smoked cigarettes, and both vaped and smoked (“dual use”) to examine the relative abuse liability of smoking versus e-cigarette use. Finally, the study compared trends between Canada, England, and the US to examine potential differences in national-level trends that may reflect different policies and product trends.

## Methods

Data were from the International Tobacco Control Policy Evaluation Project (ITC) Youth Tobacco and Vaping Study national surveys of tobacco and e-cigarette use among youth in Canada, England, and the US. The current study includes repeat cross-sectional data from eight survey waves conducted between 2017 and 2022: six annual waves and additional semiannual waves in 2020 and 2021 (see Table 1 for dates). National samples of youth aged 16 to 19 from all countries were recruited through the Nielsen Consumer Insights Global Panel (*N* = 104 467). The current analysis included a subsample of 23 145 youth who reported smoking and/or using an e-cigarette within the last 30 days. This study was reviewed and received ethics clearance through a University of Waterloo Research Ethics Committee (ORE#21847) and the King’s College London Psychiatry, Nursing, and Midwifery Research Ethics Subcommittee. A full description of the study methods is available in the Technical Reports.^32^

**Table 1.** Sample Characteristics of Youth Aged 16–19 Years Who Reported Past 30-Day Smoking and/or Vaping, by Country, Weighted % (*n*)

	2017	2018	2019	2020_Feb_	2020_Aug_	2021_Feb_	2021_Aug_	2022
Total sample (*n*)	2310	2595	2803	3523	2875	2852	2881	3308
Age, mean years (SE)								
Canada	17.6 (0.05)	17.6 (0.04)	17.7 (0.04)	17.7 (0.04)	17.6 (0.04)	17.7 (0.04)	17.7 (0.04)	17.7 (0.04)
England	17.7 (0.04)	17.7 (0.04)	17.6 (0.04)	17.7 (0.03)	17.7 (0.04)	17.7 (0.04)	17.7 (0.04)	17.6 (0.03)
USA	17.7 (0.05)	17.7 (0.04)	17.7 (0.04)	17.7 (0.04)	17.7 (0.04)	17.7 (0.05)	17.7 (0.05)	17.7 (0.05)
Sex, % (*n*)								
*Canada*								
Male	62.9 (434)	56.8 (428)	50.8 (514)	49.5 (533)	52.6 (429)	51.4 (519)	53.1 (509)	46.7 (441)
Female	37.1 (256)	43.2 (325)	49.2 (499)	50.5 (544)	47.4 (386)	48.6 (491)	46.9 (450)	53.3 (503)
*England*								
Male	54.7 (476)	58.0 (514)	52.1 (433)	52.3 (638)	56.5 (549)	53.0 (512)	51.0 (569)	51.3 (827)
Female	45.3 (394)	42.0 (372)	47.9 (397)	47.7 (581)	43.5 (422)	47.0 (454)	49.0 (548)	48.7 (785)
*USA*								
Male	55.3 (414)	55.2 (527)	51.3 (492)	53.8 (659)	52.7 (574)	43.1 (378)	50.3 (405)	50.9 (383)
Female	44.7 (334)	44.8 (428)	48.7 (468)	46.2 (567)	47.3 (515)	56.9 (498)	49.7 (401)	49.1 (369)
Race/ethnicity, % (*n*)								
*Canada*								
White (only)	72.7 (502)	51.2 (386)	61.2 (620)	63.4 (683)	64.2 (523)	66.6 (672)	63.2 (606)	61.6 (582)
Mixed/other/not stated	27.3 (189)	48.8 (367)	38.8 (393)	36.6 (395)	35.8 (291)	33.4 (337)	36.8 (353)	38.4 (362)
*England*								
White (only)	84.1 (732)	82.9 (734)	80.1 (664)	84.6 (1031)	82.5 (801)	80.9 (782)	76.9 (859)	86.4 (1392)
Mixed/other/not stated	15.9 (138)	17.1 (151)	19.9 (165)	15.4 (188)	17.5 (170)	19.1 (185)	23.1 (258)	13.6 (220)
*USA*								
White (only)	72.9 (546)	76.8 (734)	78.5 (754)	78.9 (967)	81.8 (891)	80.5 (705)	82.3 (663)	77.4 (582)
Mixed/other/not stated	27.1 (202)	23.2 (222)	21.5 (207)	21.1 (258)	18.2 (198)	19.5 (171)	17.7 (142)	22.6 (170)
Past 30-day smoking/vaping, % (*n*)								
*Exclusive vaping*								
Canada	29.3 (203)	42.4 (320)	57.0 (578)	60.2 (649)	48.5 (395)	56.6 (572)	55.9 (536)	60.1 (568)
England	19.0 (166)	17.5 (155)	29.2 (242)	22.1 (269)	24.1 (234)	25.2 (244)	34.5 (385)	37.2 (599)
USA	31.6 (237)	44.2 (422)	62.8 (603)	66.7 (817)	61.2 (666)	71.1 (623)	80.3 (647)	82.2 (619)
*Exclusive smoking*								
Canada	44.2 (305)	30.3 (228)	17.5 (177)	15.4 (166)	24.4 (199)	18.8 (190)	18.2 (174)	15.7 (148)
England	54.8 (477)	55.1 (488)	40.0 (332)	44.2 (539)	44.6 (433)	38.7 (374)	28.4 (317)	27.9 (449)
USA	31.2 (234)	24.6 (235)	13.1 (126)	10.8 (133)	15.0 (163)	11.3 (99)	6.8 (54)	5.6 (42)
*Dual use (vaping and smoking)*								
Canada	26.5 (183)	27.3 (206)	25.5 (259)	24.4 (263)	27.1 (221)	24.6 (248)	25.9 (249)	24.2 (228)
England	26.2 (228)	27.4 (243)	30.8 (256)	33.7 (411)	31.3 (304)	36.1 (349)	37.1 (415)	35.0 (564)
USA	37.2 (278)	31.2 (298)	24.1 (232)	22.5 (276)	23.9 (260)	17.6 (154)	12.9 (104)	12.2 (92)

### Measures

*Sociodemographic variables*—Sociodemographic variables include sex at birth, age (years), and race/ethnicity.^32^ Race/ethnicity was assessed using country-specific questions with multiple categories and recoded to “White (only)” or “Else” (including any other race/ethnicity not stated) to allow for cross-country comparisons.

#### Vaping and Smoking Measures

Participants were asked the following questions separately for smoking and vaping: “Have you ever tried [cigarette smoking/an e-cigarette/vaped], even one or two puffs? (Yes; No; Don’t know; Refused).” Respondents answering “Yes” were then asked the following question(s) separately for smoking and vaping: “When was the last time you [smoked a cigarette/used an e-cigarette/vaped]? (Earlier today; Not today but sometime in the past 7 days; Not in the past 7 days but sometime in the past 30 days; Not in the past 30 days but sometime in the past 6 months; Not in the past 6 months but sometime in the past 12 months; 1 to 4 years ago; 5 or more years ago; Don’t know; and Refused).” Respondents were coded according to past 30-day use: exclusive e-cigarette use, exclusive cigarette smoking, or “dual use” (both cigarette smoking and e-cigarette use).

*Frequency of use—*Respondents who reported smoking and/or using e-cigarettes in the past 30 days were asked: “In the past 30 days, on how many days did you [smoke/use an e-cigarette/vape]?,” with a numeric entry field (range: 0–30 days); “Don’t know” (15.8% for e-cigarettes, 10.7% for cigarettes) or “Refused” (0.6% for e-cigarettes, 0.4% for cigarettes) were excluded. Respondents who vaped in the past 30 days were also asked: “In the past 30 days, on the days you used an e-cigarette/vaped, how many times did you use it each day? (1 time per day; 2 to 5 times per day; 6 to 10 times per day; 11 to 20 times per day; More than 20 times per day; Don’t know; Refused).” Responses were recoded to “10 times or less” versus “More than 10 times”; “Don’t know” (3.2%) and “Refused” (0.4%) were excluded.

*Perceived addiction—*Perceived addiction is associated with more comprehensive measures of dependence for smoking and/or e-cigarette use.^11,33,34^ Respondents were asked: “Do you consider yourself addicted to [cigarettes/using e-cigarettes/vaping]? (Not at all; Yes, a little addicted; Yes, very addicted; Don’t know; Refused).” Responses were recoded as “Yes, very addicted/Yes, a little addicted” versus “Not at all”; “Don’t know” (3.5% for e-cigarettes, 1.5% for cigarettes) and “Refused” (0.5% for e-cigarettes, 0.1% for cigarettes) were excluded.

*Urges—*Experiencing strong urges/cravings to smoke and/or use an e-cigarette is a common dependence symptom reported by youth.^7,8,35,36^ Respondents were asked, “In the past 30 days, how often did you have a strong urge to [smoke/use an e-cigarette/vape]? (Several times a day; Every day or most days; At least once a week; Less than once a week; Never; Don’t know; Refused).” Responses were recoded as “Several times a day/Every day or most days” versus “less often”; “Don’t know” (2.9% for e-cigarettes, 1.1% for cigarettes) and “Refused” (0.4% for e-cigarettes, 0.2% cigarettes) were excluded.

*Time to first use after waking—*Time to first use after waking is an item on the modified Fagerström Tolerance Questionnaire (mFTQ) for adolescents and is a common indicator of nicotine dependence.^34,37^ Beginning in survey wave 3.5 (2020), respondents were asked, “How soon after waking do you [smoke your first cigarette/first use an e-cigarette/vape]? (Within 5 minutes; 6–30 minutes; 31–60 minutes; 1–4 hours (ie, in the morning); 5–8 hours (ie, in the afternoon); More than 8 hours; Don’t know; Refused).” Responses were recoded as “within 30 minutes” (“within 5 minutes” and “6–30 minutes”) versus “more than 30 minutes” (“31–60 minutes,” “1–4 hours (ie, in the morning),” “8 hours (ie, in the afternoon),” “More than 8 hours”); “Don’t know”(7.6% for e-cigarettes, 6.5% for cigarettes) and “Refused” (1.5% for e-cigarettes, 2.2% for cigarettes) were excluded.

*Vaping dependence scale—*The 4-item EDS is a modified and shortened version of the 22-item PROMIS scale created to measure e-cigarette dependence.^10^ Beginning in survey wave 3 (2019), respondents who vaped in the past 30 days were asked the following 4 items: “For each of the following statements, please choose the option that best describes you.” (1) “I find myself reaching for my e-cigarette without thinking about it.”; (2) “I drop everything to go out and get e-cigarettes or e-juice.”; (3) “I vape more before going into a situation where vaping is not allowed.”; and (4) “When I haven’t been able to vape for a few hours, the craving gets intolerable.” Never (0); Rarely (1); Sometimes (2); Often (3); Almost always (4). “Don’t know” (item 1 = 2.4%; item 2 = 1.9%; item 3 = 2.6%, item 4 = 1.9%) and “Refused” (item 1 = 0.4%; item 2 = 0.3%; item 3 = 0.4%; item 4 = 0.3%) answers were excluded. The ratings from each question were summed to calculate the total score for each respondent, with higher scores indicating greater dependence.

### Analysis

Poststratification sample weights were calculated for each country, based on age, sex, geographical region and, in the US and Canada only, trend over time for past 30-day smoking and, in the US only, race/ethnicity. In addition, subsequent survey waves were calibrated back to Wave 1 proportions for student status (student vs not) and school grades (<70%, don’t know, and refused; 70%–79%; 80%–89%; 90%–100%). Past 30-day smoking trends used for weighting were calibrated from the NYTS in the US and the Canadian Student Tobacco, Alcohol, and Drugs Survey (CSTADS) in Canada.^32^ Logistic and linear regression models (depending on the outcome examined) were fitted to examine changes in indicators of dependence between survey waves and differences between countries, adjusting for sex, age (years), race/ethnicity, and exclusive versus dual use. Within-country differences were identified by examining wave × country interaction terms for each of the dependence measures. The LSMEANS (least squares means) statement in the “proc surveylogistic” procedure was used to provide all pairwise contrasts for the country and survey wave variables. Additionally, interactions between survey wave and exclusive versus dual use were examined. “Don’t know” and “Refused” answers were excluded on a case-wise basis for each dependence measure.

## Results

### Sample Characteristics

Table 1 shows the sociodemographic profile of the sample and the prevalence of past 30-day smoking and/or vaping, by country and survey wave. Briefly, the proportion of exclusive vaping increased between 2017 and 2022 in all countries, while the proportion of exclusive smoking decreased substantially, but remained higher in England, as was the case for the proportion of dual use.

### Days of Use in the Past Month

#### Vaping

**Figure 1.**
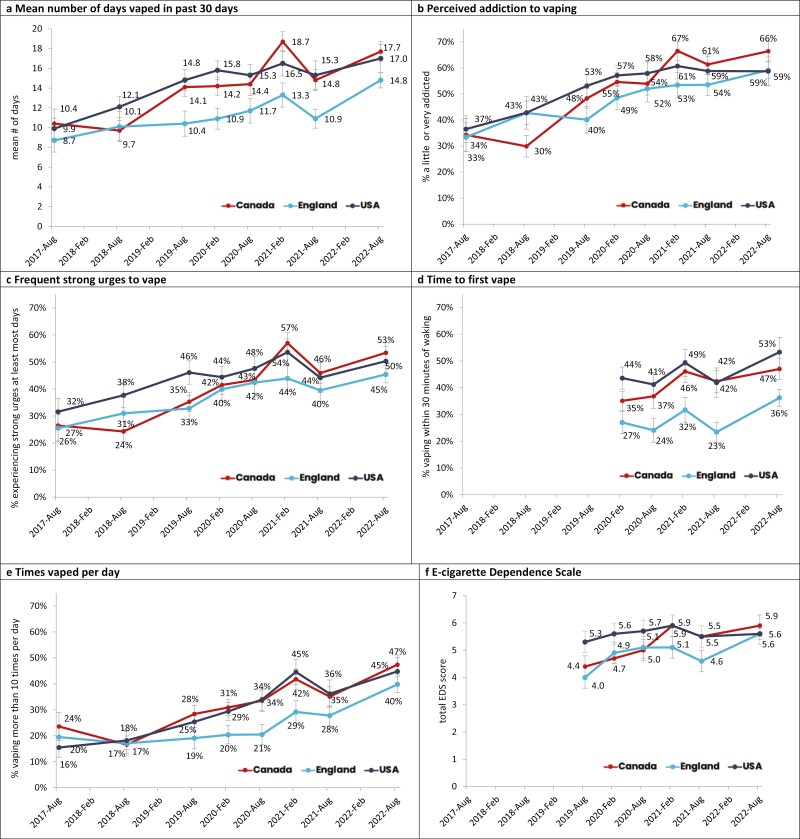
Indicators of dependence among youth aged 16–19 who had vaped in the past 30 days, 2017–2022, by country, weighted % (unless otherwise specified).

In 2022, respondents who vaped in the past 30 days reported vaping on a mean of 14.8 of the past 30 days in England versus 17.7 in Canada and 17.0 in the US (see Figure 1 and Table S1 for all estimates). The number of days vaping increased between 2017 and 2022 within all countries (Canada: 7.81, *p* < .0001; England: 6.06, *p* < .0001; US: 7.49, *p* < .0001, see Tables S3–S12 for main effect regression results). No significant interaction between survey wave and exclusive versus dual use was found (*F* = 1.89, *p* = .0664).

#### Smoking

**Figure 2.**
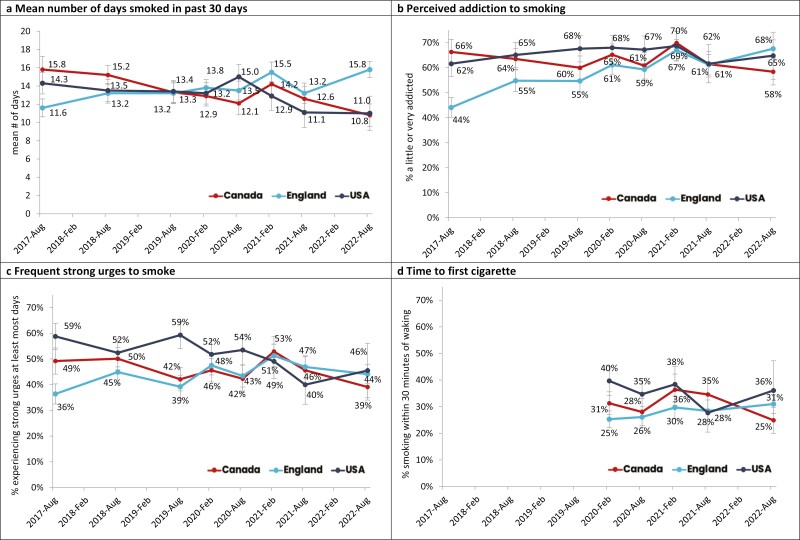
Indicators of dependence among youth aged 16–19 who had smoked in the past 30 days, 2017–2022, by country, weighted % (unless otherwise specified).

**Figure 3.**
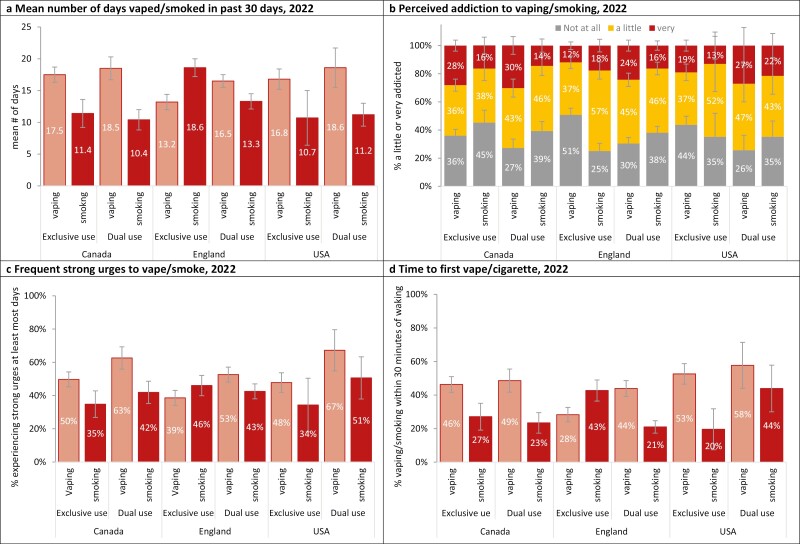
Indicators of dependence: exclusive versus dual use among youth aged 16–19 who had vaped and/or smoked in the past 30 days in 2022, by country, weighted % (unless otherwise specified).

In 2022, respondents who smoked in the past 30 days reported smoking on a mean of 15.8 of the past 30 days in England versus 10.8 in Canada and 11.0 in the US. Between 2017 and 2022, the mean number of days smoking decreased in Canada (−4.38, *p* < .0001) and the US (−3.54, *p* = .003), but increased in England (4.15, *p* < .0001, see Figure 2). An interaction between wave and exclusive versus dual use was observed (*F* = 4.47, *p* < .001), where those who “dual used” smoked on fewer days compared to those who exclusively smoked (−3.56, *p* < .0001, see Figure 3), in 2022 only.

### Perceived Addiction

#### Vaping

In 2022, 66.4% of those who used e-cigarettes in the past 30 days in Canada reported they were either a little or very addicted to e-cigarettes, compared with 59.1% in England and 58.7% in the US. Furthermore, the proportion who reported they were a little or very addicted to e-cigarettes increased between 2017 and 2022 in each of Canada (AOR = 5.03, 95% CI = 3.63 to 6.96), England (AOR = 3.39, 95% CI = 2.54 to 4.51) and the US (AOR = 3.56, 95% CI = 2.59 to 4.88).

Figure 3 shows perceived addiction among youth reporting past 30-day exclusive vaping and dual use in 2022. Across all countries, youth who reported dual use perceived themselves as more addicted to e-cigarettes than those reporting exclusive e-cigarette vaping (AOR = 2.19, 95% CI = 2.02 to 2.38); however, there was an interaction with survey wave, in which differences in e-cigarette dependence between dual use and exclusive vaping narrowed over time (*F* = 3.23, *p* = .002).

#### Smoking

In 2022, 58.4% of those who smoked in the past 30 days in Canada, 67.6% in England, and 64.8% in the US reported feeling a little or very addicted to smoking. Between 2017 and 2022, this proportion increased significantly within England (AOR = 2.43, 95% CI = 1.92 to 3.06) and decreased significantly within Canada (AOR = 0.69, 95% CI = 0.49 to 0.95), with little change in the US (AOR = 1.07, 95% CI = 0.67 to 1.70). Those who both smoked and used e-cigarettes were more likely to report feeling a little or very addicted to smoking compared to those who exclusively smoked (AOR = 1.31, 95% CI = 1.20 to 1.43). However, there was a significant interaction between survey wave and exclusive versus dual use, in which differences decreased over time (*F* = 4.34, *p* < .0001), such that, by 2022, there was no significant difference between dual and exclusive use in terms of perceived addiction to smoking (AOR = 0.79, 95% CI = 0.60 to 1.02; see Figure 2).

### Urges

#### Vaping

Among youth who used e-cigarettes within the past 30 days in 2022, 53.4% reported strong urges to vape e-cigarettes “at least most days” in Canada, compared to 45.4% in England and 50.3% in the US. Between 2017 and 2022, strong urges to use an e-cigarette “at least most days” increased among youth who reported past 30-day vaping within all three countries (Canada: AOR = 3.96, 95% CI = 2.83 to 5.53; England: AOR = 2.70, 95% CI = 2.00 to 3.65; US: AOR = 2.96, 95% CI = 2.16 to 4.06).

Experiencing strong urges to use an e-cigarette “at least most days” was greater for dual use versus exclusive e-cigarette use (AOR = 1.95, 95% CI = 1.80 to 2.11), but this effect decreased over time. The proportion of youth who exclusively used e-cigarettes reporting strong urges “at least most days” was closer to youth who “dual used” in 2022 than in 2017 (*F* = 4.01, *p* = .0002).

#### Smoking

The proportion of youth who smoked within the past 30 days experiencing strong urges to smoke “at least most days” in 2022 was 39.1% in Canada, 44.1% in England, and 45.5% in the US. Between 2017 and 2022, the proportion decreased significantly within Canada (AOR = 0.64, 95% CI = 0.47 to 0.88) and the US (AOR = 0.54, 95% CI = 0.34 to 0.86) but not England (AOR = 1.26, 95% CI = 1.00 to 1.58). There was no significant interaction between survey wave and exclusive versus dual use (*F* = 1.90, *p* = .065).

### Time to First Use

#### Vaping

In 2022, 36.2% of youth reporting past 30-day vaping in England first used an e-cigarette within 30 minutes of waking, compared with 47.0% in Canada and 53.3% in the US. The proportion of youth who vaped within 30 minutes of waking increased significantly in all countries between 2020 and 2022 (Canada: AOR = 1.67, 95% CI = 1.34 to 2.09; England: AOR = 1.57, 95% CI = 1.22 to 2.03; US: AOR = 1.55, 95% CI = 1.17 to 2.06). There was no significant interaction between survey wave and exclusive versus dual use (*F* = 0.42, *p* = .798).

#### Smoking

In 2022, the percentage of youth who smoked within the past month reported smoking within 30 minutes of waking was 24.9% in Canada, compared with 31.0% in England and 36.1% in the US. Between 2020 and 2022, no significant changes were found in Canada (AOR = 0.72, 95% CI = 0.52 to 1.01) and the US (AOR = 0.83, 95% CI = 0.49 to 1.42); however, the proportion of youth that smoked within 30 minutes of waking increased significantly in England (AOR = 1.32, 95% CI = 1.04 to 1.68). There was an interaction effect between survey wave and exclusive versus dual use, in which differences generally increased over time (*F* = 7.13, *p* < .0001): by 2022, youth who “dual used” reported smoking their first cigarette later than youth who exclusively smoked (AOR = 0.53, 95% CI = 0.40 to 0.70).

### Times Vaped Per Day

In 2022, 39.8% of youth who reported past 30-day vaping in England, compared with 44.8% in the US and 47.4% in Canada, used an e-cigarette more than 10 times per day. The proportion of youth that reported vaping more than 10 times per day increased significantly in all countries between 2017 and 2022 (Canada: AOR = 2.99, 95% CI = 2.11 to 4.22; England: AOR = 2.63, 95% CI = 1.90 to 3.64; US: AOR = 4.62, 95% CI = 3.20 to 6.68, see Table S2 for estimates). A significant interaction between survey wave and exclusive versus dual use was noted (*F* = 3.08, *p* = .003). No clear trend was found: in 2017 significantly more youth who “dual used” vaped more than 10 times per day compared to those who exclusively vaped (AOR = 1.91, 95% CI = 1.33 to 2.74); however, in 2022 there was no significant difference between the two groups (AOR = 1.19, 95% CI = 0.98 to 1.45).

### E-Cigarette Dependence Scale

In 2022, the mean scores for the 4-item EDS were 5.9 in Canada and 5.6 in both England and the US. Mean scores increased significantly between 2019 and 2022 in all countries (Canada: 1.55, *p* < .0001; England: 1.63, *p* < .0001; US: 0.60, *p* = .032). There was no significant interaction between survey wave and exclusive versus dual use (*F* = 0.61, *p* = .692).

## Discussion

Findings from the current study reveal that indicators of e-cigarette dependence among youth reporting past 30-day e-cigarette use have increased substantially between 2017 and 2022 in Canada, England, and the US. This was observed across the range of measures assessed in the study—perceived addiction, urges to use, time to first use, use events per day, and a formal dependence scale—all of which are validated indicators of vaping dependence.^38–40^; in addition, the mean number of days using an e-cigarette in the past 30 days increased by two-thirds in all three countries.

In 2017, indicators of vaping dependence were substantially lower than indicators of smoking dependence. However, by 2022, dependence measures for vaping were similar to, or greater than, those for smoking, particularly for experiencing frequent strong urges to use and use within 30 minutes of waking. This is consistent with the pharmacokinetic profile of more modern salt-based vaping products and their potential to promote and sustain dependence.^1,6^ E-cigarettes may have greater potential abuse liability given their ease and convenience of use, which can facilitate continuous vaping throughout the day. Vaping is also easier to conceal, including in indoor settings, which may increase the likelihood of more frequent use, particularly among young people.^41^

The increases in dependence measures observed in this study are consistent with findings from other studies conducted over the same period.^2,22,23,28,30^ Absolute levels of dependence reported by youth in the US in this study are slightly higher than those estimated by studies using NYTS data; however, the current study includes an older age group, and vaping dependence may be underestimated in NYTS surveys due to referring to e-cigarettes as “tobacco products.”^22–24^ In Canada, vaping estimates vary across national monitoring surveys; however, frequency of use—measured as daily vaping—has increased substantially since 2017, with national estimates of 12% daily vaping among high school students in 2021/2022.^42^ Health Canada has conducted a range of other studies that indicate similar or higher levels of vaping dependence than the current study.^43^ For example, Health Canada studies indicate that the frequency of vaping and indicators of dependence are higher among youth compared to adult smokers who vape in Canada.^43,44^ There is little evidence available from England with which to compare the current findings; however, recent ASH data have found that regular e-cigarette use among youth aged 11–17 increased from 1.2% in 2021 to 3.7% in 2023.^45^

Indicators of dependence among youth have risen in parallel with the popularity of high-nicotine, salt-based products. Younger e-cigarette users are the first to adopt emerging brands and product designs, as was the case with JUUL and the subsequent rise of disposal salt-based vapes, such as Elf Bar in the UK.^8,22,45,46^ There is a growing consensus that the chemistry of nicotine salt-based products, compared with freebase products, can increase abuse liability by facilitating the ease of nicotine inhalation and absorption.^9,22,47^ Several studies indicate a higher level of dependence among youth who use salt-based products, including earlier analyses of data from the current study.^9,28^ For example, an analysis of the 2020 NYTS found that youth who used JUUL were 77% more likely to report nicotine dependence symptoms than those using non-JUUL products.^7^ The timing of the emergence of salt-based products provides one potential explanation for the relative lag in dependence indicators in England versus Canada and the US. JUUL first emerged in 2015 in the US and in 2018 in Canada, whereas Elf Bar was the first salt-based brand to capture a substantial market share among youth in England in 2021.^8,31,45^ Additional research on the abuse liability of nicotine salt-based products is warranted in both “real world” and experimental study settings.

### Limitations

This study is subject to limitations common to survey research, such as self-report and social desirability bias; however, we expect this to be relatively consistent over time. The study sample was recruited through nonprobability sampling; therefore, the estimates may not be nationally representative. However, poststratification weights were used and the trends over time for patterns of use and indicators of dependence are highly consistent with national benchmark surveys in each country. The poststratification weights for Canada and the US were calibrated using the trend for past 30-day prevalence of smoking from national surveys, whereas this was not done for England because of insufficient sample size in national benchmark surveys to produce reliable estimates for this age group. Therefore, trends in the vaping and smoking prevalence among the England youth sample could differ from national estimates based on probability-based sampling; however, this limitation is likely to have little effect on analyses conducted on past 30-day vaping. The potential impact of the COVID-19 pandemic should also be considered. The prevalence of vaping among youth decreased during the COVID-19 pandemic in all three countries, followed by increases approach pre-pandemic highs in Canada and the US, with substantial increases in youth vaping in the UK.^17,42,45^

## Conclusions

Symptoms of e-cigarette dependence for youth vaping have increased substantially between 2017 and 2022 in Canada, England, and the US to levels that are comparable to cigarette dependence among youth who smoke. Future research should examine potential factors underlying these trends, including changes to the nicotine profile and design of e-cigarette products used by youth.

## Supplementary Material

ntae060_suppl_Supplementary_Tables_S1

ntae060_suppl_Supplementary_Tables_S2

ntae060_suppl_Supplementary_Tables_S3-S12

ntae060_suppl_Supplementary_Tables_S13-S15

ntae060_suppl_Supplementary_Tables_S16

ntae060_suppl_Supplementary_Tables_S17

## Data Availability

Deidentified study data may be made available on request to researchers who submit a proposal that is approved by the principal investigator. Proposals should be submitted to David Hammond (dhammond@uwaterloo.ca).
