# Supplementary material for: Comparison of Indicators of Dependence for Vaping and Smoking: Trends Between 2017 and 2022 Among Youth in Canada, England, and the United States
Source: Nicotine Tob Res. 2024 Mar 26;26(9):1192–200. doi: 10.1093/ntr/ntae060 (PMC11339172; doi:10.1093/ntr/ntae060)
Supplement: ntae060_suppl_Supplementary_Tables_S1 [file ntae060_suppl_supplementary_tables_s1.pdf]

Supplementary Table S1. Indicators of dependence among youth aged 16-19 who report past 30-day smoking and vaping, 2017-2022, by country, weighted % (n)

	2017	2018	2019	2020a	2020b	2021a	2021b	2022
**Perceive self as "very" or "a little addicted";** %(n)								
**Vaping**								
Canada	34.3 (123)	29.9 (152)	48.3 (391)	54.6 (490)	53.9 (326)	66.5 (517)	61.3 (453)	66.4 (502)
England	33.4 (119)	42.7 (162)	40.1 (193)	48.5 (322)	52.0 (270)	53.4 (303)	53.5 (412)	59.1 (654)
USA	36.5 (184)	42.8 (301)	53.1 (428)	57.1 (610)	57.9 (515)	60.7 (453)	58.8 (417)	58.7 (392)
**Smoking**								
Canada	66.3 (315)	63.5 (271)	60.0 (257)	65.2 (275)	60.8 (252)	69.9 (303)	61.4 (253)	58.4 (214)
England	44.1 (301)	54.8 (401)	54.7 (317)	61.1 (573)	59.3 (431)	67.0 (472)	61.2 (442)	67.6 (673)
USA	61.6 (311)	65.1 (340)	67.6 (239)	68.0 (275)	67.2 (281)	68.7 (168)	61.6 (96)	64.8 (86)
**Strong urges at least most days;** %(n)								
**Vaping**								
Canada	26.5 (97)	24.3 (124)	35.3 (287)	41.5 (366)	43.4 (259)	57.0 (446)	45.9 (343)	53.4 (413)
England	25.5 (95)	31.0 (120)	32.8 (159)	39.9 (267)	42.4 (220)	43.9 (250)	39.5 (304)	45.4 (513)
USA	31.6 (160)	37.7 (262)	46.1 (370)	44.4 (476)	47.6 (424)	53.6 (407)	44.2 (316)	50.3 (346)
**Smoking**								
Canada	49.2 (237)	50.1 (214)	42.1 (181)	45.6 (193)	42.3 (176)	52.9 (231)	45.6 (190)	39.1 (144)
England	36.4 (251)	44.9 (326)	39.2 (228)	47.5 (446)	43.4 (313)	51.3 (367)	46.9 (340)	44.1 (442)
USA	58.8 (297)	52.4 (276)	59.3 (210)	51.8 (208)	53.5 (225)	49.1 (121)	40.0 (63)	45.5 (60)
**First vape/cigarette within 30 mins of waking;** %(n)								
**Vaping**								
Canada	-	-	-	35.1 (281)	36.8 (205)	46.1 (352)	42.4 (294)	47.0 (343)
England	-	-	-	27.0 (164)	24.1 (118)	31.7 (171)	23.4 (165)	36.2 (383)
USA	-	-	-	43.6 (437)	41.2 (357)	49.3 (353)	41.9 (287)	53.3 (351)
**Smoking**								
Canada	-	-	-	31.3 (124)	28.0 (108)	36.4 (148)	34.6 (129)	24.9 (80)
England	-	-	-	25.3 (220)	26.1 (174)	29.7 (195)	28.4 (188)	31.0 (290)
USA	-	-	-	39.7 (151)	34.7 (139)	38.4 (90)	27.7 (39)	36.1 (43)
**Days of use in past 30 days;** mean (SE)								
**Vaping**								
Canada	10.4 (0.7)	9.7 (0.5)	14.1 (0.5)	14.2 (0.5)	14.4 (0.6)	18.7 (0.5)	14.8 (0.5)	17.7 (0.5)
England	8.7 (0.6)	10.1 (0.8)	10.4 (0.6)	10.9 (0.5)	11.7 (0.6)	13.3 (0.6)	10.9 (0.5)	14.8 (0.4)
USA	9.9 (0.5)	12.1 (0.5)	14.8 (0.5)	15.8 (0.5)	15.3 (0.5)	16.5 (0.7)	15.3 (0.7)	17.0 (0.7)
**Smoking**								
Canada	15.8 (0.7)	15.2 (0.5)	13.3 (0.6)	12.9 (0.6)	12.1 (0.6)	14.2 (0.7)	12.6 (0.7)	10.8 (0.6)
England	11.6 (0.5)	13.2 (0.6)	13.2 (0.6)	13.8 (0.5)	13.5 (0.5)	15.5 (0.6)	13.2 (0.6)	15.8 (0.4)
USA	14.3 (0.6)	13.5 (0.6)	13.4 (0.6)	13.2 (0.6)	15.0 (0.7)	12.9 (0.8)	11.1 (0.8)	11.0 (1.0)
