# Supplementary material for: Comparison of Indicators of Dependence for Vaping and Smoking: Trends Between 2017 and 2022 Among Youth in Canada, England, and the United States
Source: Nicotine Tob Res. 2024 Mar 26;26(9):1192–200. doi: 10.1093/ntr/ntae060 (PMC11339172; doi:10.1093/ntr/ntae060)
Supplement: ntae060_suppl_Supplementary_Tables_S2 [file ntae060_suppl_supplementary_tables_s2.pdf]

Supplementary Table S2. Vaping dependence measures among youth aged 16-19 who report past 30-day vaping, 2017-2022, by country, weighted % (n)

	2017	2018	2019	2020a	2020b	2021a	2021b	2022
**Vaping more than 10 times per day**								
Canada	23.6% (87)	16.6% (85)	28.4% (227)	30.9% (272)	33.5% (200)	41.8% (332)	35.1% (263)	47.4% (363)
England	19.5% (72)	17.1% (66)	19.1% (90)	20.4% (133)	20.5% (107)	29.2% (168)	27.8% (214)	39.8% (451)
US	15.5% (77)	18.2% (125)	25.4% (205)	29.4% (317)	34.0% (306)	44.6% (334)	36.2% (261)	44.8%- (304)
**E-cigarette Dependence Scale; mean score (SE)**								
"I find myself reaching for my e-cigarette without thinking about it."								
Canada	-	-	1.5 (0.05)	1.5 (0.05)	1.6 (0.06)	2.0 (0.05)	1.7 (0.05)	1.8 (0.05)
England	-	-	1.1 (0.06)	1.4 (0.06)	1.5 (0.06)	1.6 (0.06)	1.3 (0.05)	1.7 (0.04)
US	-	-	1.7 (0.05)	1.7 (0.05)	1.7 (0.06)	1.8 (0.07)	1.7 (0.07)	1.8 (0.07)
"I drop everything to go out and get e-cigarettes or e-juice."								
Canada	-	-	0.7 (0.04)	0.8 (0.04)	0.9 (0.05)	1.0 (0.05)	1.0 (0.04)	1.1 (0.05)
England	-	-	0.8 (0.06)	0.9 (0.05)	1.1 (0.06)	0.9 (0.05)	0.9 (0.05)	1.0 (0.04)
US	-	-	1.0 (0.05)	1.0 (0.04)	1.1 (0.05)	1.0 (0.06)	0.9 (0.06)	1.0 (0.07)
"I vape more before going into a situation where vaping is not allowed."								
Canada	-	-	1.4 (0.05)	1.5 (0.05)	1.5 (0.06)	1.8 (0.06)	1.6 (0.05)	1.7 (0.05)
England	-	-	1.1 (0.06)	1.5 (0.06)	1.4 (0.07)	1.6 (0.06)	1.4 (0.06)	1.7 (0.04)
US	-	-	1.6 (0.06)	1.8 (0.06)	1.7 (0.06)	1.8 (0.07)	1.8 (0.08)	1.8 (0.07)
"When I haven't been able to vape for a few hours, the craving gets intolerable."								
Canada	-	-	0.9 (0.04)	1.0 (0.04)	1.0 (0.05)	1.2 (0.05)	1.2 (0.05)	1.3 (0.05)
England	-	-	0.9 (0.06)	1.2 (0.06)	1.1 (0.06)	1.1 (0.06)	1.0 (0.05)	1.2 (0.04)
US	-	-	1.1 (0.05)	1.2 (0.05)	1.3 (0.06)	1.3 (0.06)	1.2 (0.07)	1.2 (0.07)
Total Score of 4-item EDS								
Canada	-	-	4.4 (0.2)	4.7 (0.1)	5.0 (0.2)	5.9 (0.2)	5.5 (0.2)	5.9 (0.2)
England	-	-	4.0 (0.2)	4.9 (0.2)	5.1 (0.2)	5.1 (0.2)	4.6 (0.2)	5.6 (0.1)
US	-	-	5.3 (0.2)	5.6 (0.2)	5.7 (0.2)	5.9 (0.2)	5.5 (0.2)	5.6 (0.2)
