# Supplementary material for: Comparison of Indicators of Dependence for Vaping and Smoking: Trends Between 2017 and 2022 Among Youth in Canada, England, and the United States
Source: Nicotine Tob Res. 2024 Mar 26;26(9):1192–200. doi: 10.1093/ntr/ntae060 (PMC11339172; doi:10.1093/ntr/ntae060)
Supplement: ntae060_suppl_Supplementary_Tables_S3-S12 [file ntae060_suppl_supplementary_tables_s3-s12.pdf]

Supplementary Table S3. Linear regression of frequency (# of days) of e-cigarette use within the last 30 days (n=13847)

	Beta	Standard error	95% CI (lower, upper)	P level
**Country**				
US (ref) vs England	-3.72	0.29	-4.29, -3.15	<.0001
Canada (ref) vs England	-3.74	0.28	-4.28, -3.19	<.0001
Canada (ref) vs US	-0.02	0.28	-0.56, 0.53	0.949
**Survey wave**				
2017 (ref) vs. 2018	1.12	0.49	0.15, 2.09	0.022
2018 (ref) vs. 2019	2.77	0.47	1.86, 3.68	<.0001
2019 (ref) vs. 2020a	0.43	0.42	-0.40, 1.27	0.305
2020a (ref) vs. 2020b	0.01	0.44	-0.86, 0.88	0.989
2020b (ref) vs. 2021a	2.46	0.48	1.51, 3.40	<.0001
2021a (ref) vs. 2021b	-2.48	0.49	-3.42, -1.54	<.0001
2021b (ref) vs. 2022	2.98	0.45	2.10, 3.86	<.0001
**Age** (years)	1.05	0.11	0.84, 1.26	<.0001
**Race/ethnicity** (ref=white)	-2.91	0.24	-3.39, -2.44	<.0001
**Sex** (ref= female)	0.68	0.23	0.22, 1.14	0.004
**Exclusive vs. dual use** (ref= exclusive)	1.19	0.23	0.74, 1.65	<.0001

Supplementary Table S4. Linear regression of frequency (# of days) of smoking within the last 30 days (n= 11,552)

	Beta	Standard error	95% CI (lower, upper)	P level
**Country**				
US (ref) vs England	0.18	0.32	-0.46, 0.82	0.580
Canada (ref) vs England	-0.17	0.31	-0.77, 0.43	0.570
Canada (ref) vs US	-0.35	0.34	-1.03, 0.32	0.305
**Survey wave**				
2017 (ref) vs. 2018	0.38	0.49	-0.57, 1.34	0.431
2018 (ref) vs. 2019	-0.50	0.49	-1.45, 0.45	0.303
2019 (ref) vs. 2020a	0.07	0.47	-0.85, 0.98	0.889
2020a (ref) vs. 2020b	0.05	0.47	-0.87, 0.97	0.915
2020b (ref) vs. 2021a	1.12	0.53	0.08, 2.16	0.035
2021a (ref) vs. 2021b	-1.85	0.55	-2.95, -0.76	<0.001
2021b (ref) vs. 2022	1.32	0.53	0.28, 2.37	0.013
**Age** (years)	0.09	0.12	-0.15, 0.32	0.473
**Ethnicity** (ref=white)	-2.35	0.28	-2.91, -1.79	<.0001
**Sex** (ref=female)	2.03	0.24	1.55, 2.51	<.0001
**Exclusive vs. dual use** (ref= exclusive)	-0.43	0.26	-0.93, 0.07	0.093

Supplementary Table S5. Logistic regression on self-reporting being "very" or "a little" addicted to e-cigarettes (n= 16,190)

	Beta	Odds ratio	95% CI (lower, upper)	P level
**Country**				
US (ref) vs England	-0.49	0.61	0.55, 0.68	<.0001
Canada (ref) vs England	-0.43	0.65	0.59, 0.72	<.0001
Canada (ref) vs US	0.07	1.07	0.97, 1.17	0.165
**Survey wave**				
2017 (ref) vs. 2018	0.22	1.24	1.03, 1.50	0.023
2018 (ref) vs. 2019	0.52	1.68	1.43, 1.97	<.0001
2019 (ref) vs. 2020a	0.23	1.25	1.09, 1.44	0.002
2020a (ref) vs. 2020b	0.02	1.03	0.88, 1.19	0.743
2020b (ref) vs. 2021a	0.30	1.35	1.15, 1.59	<0.001
2021a (ref) vs. 2021b	-0.10	0.91	0.78, 1.06	0.228
2021b (ref) vs. 2022	0.20	1.23	1.06, 1.42	0.008
**Age** (years)	0.08	1.08	1.04, 1.12	<.0001
**Ethnicity** (ref= white)	-0.18	0.84	0.77, 0.91	<.0001
**Sex** (ref= female)	0.31	1.37	1.26, 1.48	<.0001
**Exclusive vs. dual use** (ref= exclusive)	0.79	2.19	2.02, 2.38	<.0001

Supplementary Table S6. Logistic regression on self-reporting being "very" or "a little" addicted to cigarettes (n= 13,043)

	Beta	Odds ratio	95% CI (lower, upper)	P level
**Country**				
US (ref) vs England	-0.24	-0.79	0.70, 0.88	<.0001
Canada (ref) vs England	-0.15	0.86	0.78, 0.95	0.003
Canada (ref) vs US	0.09	1.09	0.97, 1.24	0.147
**Survey wave**				
2017 (ref) vs. 2018	0.17	1.18	1.01, 1.39	0.040
2018 (ref) vs. 2019	-0.03	0.97	0.82, 1.14	0.693
2019 (ref) vs. 2020a	1.20	1.22	1.04, 1.43	0.017
2020a (ref) vs. 2020b	-0.11	0.90	0.77, 1.06	0.202
2020b (ref) vs. 2021a	0.30	1.36	1.13, 1.63	0.001
2021a (ref) vs. 2021b	-0.31	0.73	0.61, 0.88	0.002
2021b (ref) vs. 2022	0.18	1.20	1.00, 1.43	0.051
**Age** (years)	-0.07	0.93	0.90, 0.97	<.001
**Ethnicity** (ref= white)	-0.02	0.98	0.89, 1.08	0.690
**Sex** (ref= female)	0.42	1.52	1.40, 1.65	<.0001
**Exclusive vs. dual use** (ref= exclusive)	0.27	1.31	1.20, 1.43	<.0001

Supplementary Table S7. Logistic regression on strong urges to use e-cigarettes (n=16,312)

	Beta	Odds ratio	95% CI (lower, upper)	P level
**Country**				
US (ref) vs England	-0.51	0.60	0.54, 0.66	<.0001
Canada (ref) vs England	-0.37	0.69	0.63, 0.76	<.0001
Canada (ref) vs US	0.14	1.15	1.05, 1.27	0.002
**Survey wave**				
2017 (ref) vs. 2018	0.22	1.25	1.03, 1.52	0.028
2018 (ref) vs. 2019	0.41	1.51	1.28, 1.77	<.0001
2019 (ref) vs. 2020a	0.13	1.14	0.99, 1.31	0.077
2020a (ref) vs. 2020b	0.09	1.09	0.94, 1.27	0.233
2020b (ref) vs. 2021a	0.36	1.43	1.22, 1.67	<.0001
2021a (ref) vs. 2021b	-0.33	0.72	0.61, 0.84	<.0001
2021b (ref) vs. 2022	0.29	1.34	1.16, 1.55	<.0001
**Age** (years)	0.08	1.09	1.05, 1.13	<.0001
**Ethnicity** (ref= white)	-0.25	0.78	0.71, 0.85	<.0001
**Sex** (ref= female)	0.22	1.24	1.15, 1.34	<.0001
**Exclusive vs. dual use** (ref= exclusive)	0.67	1.95	1.80, 2.11	<.0001

Supplementary Table S8. Logistic regression on strong urges to smoke (n= 13,081)

	Beta	Odds ratio	95% CI (lower, upper)	P level
**Country**				
US (ref) vs England	-0.27	0.76	0.68, 0.85	<.0001
Canada (ref) vs England	-0.05	0.95	0.86, 1.05	0.345
Canada (ref) vs US	0.23	1.25	1.12, 1.41	<.001
**Survey wave**				
2017 (ref) vs. 2018	0.06	1.06	0.90, 1.24	0.490
2018 (ref) vs. 2019	-0.14	0.87	0.74, 1.02	0.080
2019 (ref) vs. 2020a	0.12	1.13	0.97, 1.32	0.115
2020a (ref) vs. 2020b	-0.11	0.90	0.77, 1.05	0.179
2020b (ref) vs. 2021a	0.26	1.29	1.08, 1.54	0.004
2021a (ref) vs. 2021b	-0.24	0.79	0.66, 0.95	0.011
2021b (ref) vs. 2022	-0.11	0.90	0.76, 1.07	0.234
**Age** (years)	-0.08	0.93	0.89, 0.96	<.001
**Ethnicity** (ref= white)	-0.11	0.89	0.81, 0.98	0.021
**Sex** (ref= female)	0.29	1.34	1.24, 1.45	<.0001
**Exclusive vs. dual use** (ref= exclusive)	0.29	1.33	1.22, 1.45	<.0001

Supplementary Table S9. Logistic regression on first e-cigarette/vape within 30 minutes of waking (n= 10,938)

	Beta	Odds ratio	95% CI (lower, upper)	P level
**Country**				
US (ref) vs England	-0.89	0.41	0.36, 0.47	<.0001
Canada (ref) vs England	-0.71	0.49	0.44, 055	<.0001
Canada (ref) vs US	0.18	1.20	1.07, 1.34	0.002
**Survey wave**				
2017 (ref) vs. 2018	-	-	-	-
2018 (ref) vs. 2019	-	-	-	-
2019 (ref) vs. 2020a	-	-	-	-
2020a (ref) vs. 2020b	-0.06	0.94	0.81, 1.11	0.472
2020b (ref) vs. 2021a	0.37	1.45	1.23, 1.72	<.0001
2021a (ref) vs. 2021b	-0.26	0.77	0.66, 0.91	0.002
2021b (ref) vs 2022	0.42	1.52	1.31, 1.78	<.0001
**Age** (years)	0.16	1.18	1.12, 1.23	<.0001
**Ethnicity** (ref= white)	-0.33	0.72	0.65, 0.81	<.0001
**Sex** (ref= female)	-0.09	0.92	0.83, 1.01	0.083
**Exclusive vs. dual use** (ref= exclusive)	0.36	1.43	1.29, 1.59	<.0001

Supplementary Table S10. Logistic regression on the first cigarette within 30 minutes of waking (n=7,609)

	**Beta**	**Odds ratio**	**95% CI (lower, upper)**	**P level**
**Country**				
US (ref) vs England	-0.38	0.69	0.58, 0.81	<.0001
Canada (ref) vs England	-0.17	0.84	0.73, 0.97	0.019
Canada (ref) vs US	0.21	1.23	1.04, 1.46	0.017
**Survey wave**				
2017 (ref) vs. 2018	-	-	-	-
2018 (ref) vs. 2019	-	-	-	-
2019 (ref) vs. 2020a	-	-	-	-
2020a (ref) vs. 2020b	-0.08	0.92	0.77, 1.10	0.354
2020b (ref) vs. 2021a	0.24	1.27	1.05, 1.54	0.016
2021a (ref) vs. 2021b	-0.12	0.88	0.72, 1.08	0.227
2021b (ref) vs 2022	0.02	1.02	0.83, 1.24	0.879
**Age** (years)	0.05	1.05	0.99, 1.11	0.096
**Ethnicity** (ref= white)	-0.12	0.89	0.77, 1.02	0.100
**Sex** (ref= female)	0.27	1.31	1.17, 1.47	<.0001
**Exclusive vs. dual use** (ref= exclusive)	0.01	1.01	0.90, 1.14	0.846

Supplementary Table S11. Logistic regression on the daily use of e-cigarettes of more than 10 times per day (n= 16,281)

	Beta	Odds ratio	95% CI (lower, upper)	P level
**Country**				
US (ref) vs England	-0.39	0.68	0.61, 0.75	<.0001
Canada (ref) vs England	-049	0.62	0.56, 0.68	<.0001
Canada (ref) vs US	-0.09	0.91	0.83, 1.00	0.058
**Survey wave**				
2017 (ref) vs. 2018	-0.13	0.88	0.69, 1.11	0.273
2018 (ref) vs. 2019	0.47	1.60	1.31, 1.95	<.0001
2019 (ref) vs. 2020a	0.12	1.13	0.97, 1.32	0.121
2020a (ref) vs. 2020b	0.15	1.16	0.99, 1.36	0.072
2020b (ref) vs. 2021a	0.39	1.47	1.25, 1.73	<.0001
2021a (ref) vs. 2021b	-0.23	0.80	0.68, 0.93	0.005
2021b (ref) vs 2022	0.48	1.61	1.39, 1.87	<.0001
**Age** (years)	0.26	1.30	1.25, 1.35	<.0001
**Ethnicity** (ref= white)	-0.40	0.67	0.61, 0.74	<.0001
**Sex** (ref= female)	-0.18	0.83	0.76, 0.91	<.0001
**Exclusive vs. dual use** (ref= exclusive)	0.11	1.11	1.02, 1.21	0.013

Supplemental Table S12. Linear regression of E-cigarette Dependence Scale (n= 14,059)

	Beta	Standard error	95% CI (lower, upper)	P level
**Country**				
US (ref) vs England	-1.38	0.11	-1.5, -1.17	<.0001
Canada (ref) vs England	-0.87	0.10	-1.0, -0.68	<.0001
Canada (ref) vs US	0.51	0.10	0.31, 0.71	<.0001
**Survey wave**				
2017 (ref) vs. 2018	-	-	-	-
2018 (ref) vs. 2019	-	-	-	-
2019 (ref) vs. 2020a	0.45	0.14	0.18, 0.71	0.001
2020a (ref) vs. 2020b	0.16	0.15	-0.13, 0.45	0.280
2020b (ref) vs. 2021a	0.48	0.15	0.18, 0.79	0.002
2021a (ref) vs. 2021b	-0.39	0.15	-0.89, -0.09	0.010
2021b (ref) vs 2022	0.59	0.15	0.30,0.87	<.0001
**Age** (years)	0.20	0.04	0.13, 0.28	<.0001
**Ethnicity** (ref=white)	-0.41	0.09	-0.59, -0.23	<.0001
**Sex** (ref=female)	0.25	0.09	0.09, 0.42	0.003
**Exclusive vs. dual use** (ref= exclusive)	1.99	0.09	1.82, 2.16	<.0001
