# Supplementary material for: Comparison of Indicators of Dependence for Vaping and Smoking: Trends Between 2017 and 2022 Among Youth in Canada, England, and the United States
Source: Nicotine Tob Res. 2024 Mar 26;26(9):1192–200. doi: 10.1093/ntr/ntae060 (PMC11339172; doi:10.1093/ntr/ntae060)
Supplement: ntae060_suppl_Supplementary_Tables_S13-S15 [file ntae060_suppl_supplementary_tables_s13-s15.pdf]

Supplemental Table S13. Youth self-reporting being very addicted to e-cigarettes/cigarettes, by country, weighted %/n

	2017	2018	2019	2020a	2020b	2021a	2021b	2022
**Canada**								
Exclusive use								
Vaping	5.0% (9)	3.6% (11)	10.9% (61)	14.7% (94)	13.5% (52)	21.3% (115)	20.4% (103)	28.1% (153)
Smoking	19.9% (60)	20.3% (45)	22.4% (39)	20.3% (33)	17.9% (36)	27.0% (51)	24.1% (41)	16.4% (23)
Dual use								
Vaping	18.2% (31)	15.7% (31)	17.9% (45)	21.6% (56)	21.1% (46)	32.6% (78)	23.3% (55)	30.3% (64)
Smoking	16.3% (29)	28.7% (59)	18.8% (48)	14.3% (37)	15.3% (33)	20.4% (50)	19.0% (46)	14.4% (32)
**England**								
Exclusive use								
Vaping	8.2% (13)	4.7% (7)	5.8% (14)	8.1% (21)	9.5% (21)	9.3% (22)	11.5% (42)	11.8% (68)
Smoking	9.4% (43)	14.3% (70)	13.5% (44)	14.8% (79)	13.0% (55)	17.5% (64)	15.8% (49)	17.7% (78)
Dual use								
Vaping	18.3% (38)	13.5% (31)	11.5% (28)	10.6% (43)	12.5% (36)	14.7% (49)	19.5% (79)	24.3% (131)
Smoking	17.5% (39)	15.1% (37)	15.8% (40)	18.1% (74)	15.6% (47)	14.2% (48)	14.8% (60)	16.3% (91)
**US**								
Exclusive use								
Vaping	0.9% (2)	6.6% (27)	8.8% (51)	15.7% (125)	17.5% (111)	20.9% (125)	20.8% (126)	19.0% (110)
Smoking	12.9% (29)	14.7% (34)	15.4% (19)	21.1% (28)	17.7% (28)	26.9% (26)	15.0% (8)	13.0% (5)
Dual use								
Vaping	18.6% (50)	22.8% (66)	25.3% (57)	25.2% (68)	29.0% (73)	23.6% (35)	27.4% (28)	27.1% (24)
Smoking	15.6% (43)	22.0% (64)	20.9% (48)	18.5% (50)	25.5% (66)	18.6% (28)	16.0% (17)	21.5% (19)

Supplementary Table S14. Youth self-reporting being a little addicted to e-cigarettes/cigarettes, by country, weighted %/n

	2017	2018	2019	2020a	2020b	2021a	2021b	2022
**Canada**								
Exclusive use								
Vaping	14.8% (28)	17.3% (54)	32.4% (180)	33.2% (212)	34.2% (132)	41.9% (225)	36.0% (181)	35.9% (195)
Smoking	46.4% (139)	44.0% (97)	36.4% (64)	41.9% (68)	42.8% (85)	41.7% (78)	40.6% (70)	38.4% (55)
Dual use								
Vaping	32.6% (55)	28.5% (56)	41.4% (105)	49.5% (128)	43.9% (96)	41.4% (99)	48.6% (114)	42.5% (90)
Smoking	50.0% (87)	34.1% (70)	42.0% (107)	52.8% (137)	45.4% (98)	50.4% (124)	40.1% (96)	46.3% (103)
**England**								
Exclusive use								
Vaping	16.0% (24)	26.6% (39)	24.3% (57)	35.0% (92)	33.7% (76)	28.0% (65)	29.5% (108)	37.3% (213)
Smoking	26.4% (122)	34.2% (167)	35.0% (114)	40.3% (214)	41.9% (179)	45.9% (167)	44.3% (138)	57.2% (251)
Dual use								
Vaping	21.8% (45)	36.3% (84)	38.0% (94)	41.4% (166)	46.5% (135)	50.0% (167)	45.3% (183)	45.3% (243)
Smoking	44.0% (97)	52.4% (127)	46.9% (119)	50.8% (207)	50.0% (150)	56.6% (193)	47.4% (194)	45.6% (254)
**US**								
Exclusive use								
Vaping	17.9% (42)	24.6% (101)	39.5% (230)	36.8% (294)	35.2% (224)	37.0% (221)	36.7% (222)	37.3% (216)
Smoking	42.1% (96)	44.8% (104)	46.7% (58)	48.0% (63)	45.6% (73)	37.6% (36)	46.0% (24)	51.8% (22)
Dual use								
Vaping	33.5% (90)	36.4% (106)	40.3% (90)	45.5% (123)	42.3% (106)	48.5% (71)	39.7% (41)	47.2% (42)
Smoking	51.6% (142)	47.6% (138)	49.8% (114)	49.0% (134)	44.1% (114)	52.8% (79)	45.9% (48)	43.3% (39)

Supplementary Table S15. Youth self-reporting being not at all addicted to e-cigarettes/cigarettes, by country, weighted %/n

	**2017**	**2018**	**2019**	**2020a**	**2020b**	**2021a**	**2021b**	**2022**
**Canada**								
Exclusive use								
Vaping	80.2% (153)	79.1% (248)	56.7% (316)	52.1% (331)	52.3% (202)	36.8% (198)	43.7% (220)	36.0% (196)
Smoking	33.7% (101)	35.8% (79)	41.2% (72)	37.8% (62)	39.2% (78)	31.3% (59)	35.3% (61)	45.3% (65)
Dual use								
Vaping	49.2% (83)	55.9% (110)	40.6% (103)	28.9% (75)	35.1% (76)	26.0% (62)	28.1% (66)	27.3% (58)
Smoking	33.7% (59)	37.2% (76)	39.2% (100)	32.9% (85)	39.2% (85)	29.2% (72)	40.9% (98)	39.3% (88)
**England**								
Exclusive use								
Vaping	75.8% (115)	68.7% (101)	69.8% (164)	56.9% (150)	56.9% (129)	62.7% (146)	59.1% (216)	50.8% (290)
Smoking	64.2% (296)	51.4% (251)	51.5% (168)	44.9% (238)	45.1% (193)	36.6% (133)	40.0% (125)	25.1% (110)
Dual use								
Vaping	59.9% (123)	50.2% (116)	50.4% (124)	48.0% (192)	41.1% (120)	35.3% (118)	35.2% (142)	30.4% (163)
Smoking	38.4% (85)	32.6% (79)	37.3% (95)	31.1% (127)	34.4% (103)	29.2% (100)	37.9% (155)	38.1% (212)
**US**								
Exclusive use								
Vaping	81.2% (191)	68.8% (284)	51.7% (301)	47.5% (379)	47.4% (302)	42.1% (251)	42.6% (258)	43.7% (253)
Smoking	45.0% (103)	40.5% (94)	37.9% (47)	30.9% (41)	36.8% (59)	35.6% (34)	39.0% (20)	35.2% (15)
Dual use								
Vaping	47.9% (129)	40.8% (119)	34.4% (77)	29.3% (79)	28.7% (72)	27.8% (41)	32.9% (34)	25.7% (23)
Smoking	32.9% (91)	30.3% (88)	29.4% (67)	32.5% (89)	30.4% (78)	28.6% (43)	38.1% (40)	25.2% (32)
