# Supplementary material for: Comparison of Indicators of Dependence for Vaping and Smoking: Trends Between 2017 and 2022 Among Youth in Canada, England, and the United States
Source: Nicotine Tob Res. 2024 Mar 26;26(9):1192–200. doi: 10.1093/ntr/ntae060 (PMC11339172; doi:10.1093/ntr/ntae060)
Supplement: ntae060_suppl_Supplementary_Tables_S16 [file ntae060_suppl_supplementary_tables_s16.pdf]

Supplementary Table S16. Frequencies by dual vs. exclusive use of significant wave*exclusive vs dual interactions for past 30-day vaping.

	**2017**	**2018**	**2019**	**2020a**	**2020b**	**2021a**	**2021b**	**2022**
**Perceived addiction**								
Exclusive vaping	20.1%	27.5%	43.2%	49.3%	49.4%	56.5%	53.0%	56.4%
Dual using	47.9%	52.1%	58.0%	62.7%	64.7%	69.3%	67.4%	70.9%
**Strong Urges**								
Exclusive vaping	16.7%	21.5%	34.5%	37.5%	40.5%	49.7%	39.1%	45.3%
Dual using	38.6%	44.2%	47.1%	50.8%	52.4%	57.2%	51.4%	56.7%
**Times vaped per day**								
Exclusive vaping	14.3%	16.5%	24.4%	28.9%	32.6%	41.4%	32.4%	42.8%
Dual using	23.4%	16.6%	26.6%	25.5%	26.9%	35.5%	34.2%	44.6%
