# Supplementary material for: Comparison of Indicators of Dependence for Vaping and Smoking: Trends Between 2017 and 2022 Among Youth in Canada, England, and the United States
Source: Nicotine Tob Res. 2024 Mar 26;26(9):1192–200. doi: 10.1093/ntr/ntae060 (PMC11339172; doi:10.1093/ntr/ntae060)
Supplement: ntae060_suppl_Supplementary_Tables_S17 [file ntae060_suppl_supplementary_tables_s17.pdf]

Supplementary Table S17. Frequencies by dual vs. exclusive use of significant wave*exclusive vs dual interactions for past 30-day smoking.

	**2017**	**2018**	**2019**	**2020a**	**2020b**	**2021a**	**2021b**	**2022**
**Perceived addiction**								
Exclusive smoking	49.5%	55.0%	54.1%	58.8%	58.1%	65.1%	61.6%	69.6%
Dual using	65.1%	67.0%	64.5%	68.0%	65.6%	70.9%	61.1%	61.9%
**Time to first use**								
Exclusive smoking	-	-	-	28.9%	27.3%	30.1%	26.2%	37.8%
Dual using	-	-	-	31.0%	30.7%	36.2%	33.2%	23.9%
**Mean #days**								
Exclusive smoking	13.3	13.3	12.9	13.9	13.6	15.3	13.2	16.6
Dual using	14.0	14.5	13.7	13.0	13.5	14.0	12.5	12.4
